# Supplementary material for: Spin-momentum locked interaction between guided photons and surface electrons in topological insulators
Source: Nat Commun. 2017 Dec 15;8:2141. doi: 10.1038/s41467-017-02264-y (PMC5732163; doi:10.1038/s41467-017-02264-y)
Supplement: Supplementary file 1 — Supplementary Information [file 41467_2017_2264_MOESM1_ESM.pdf]

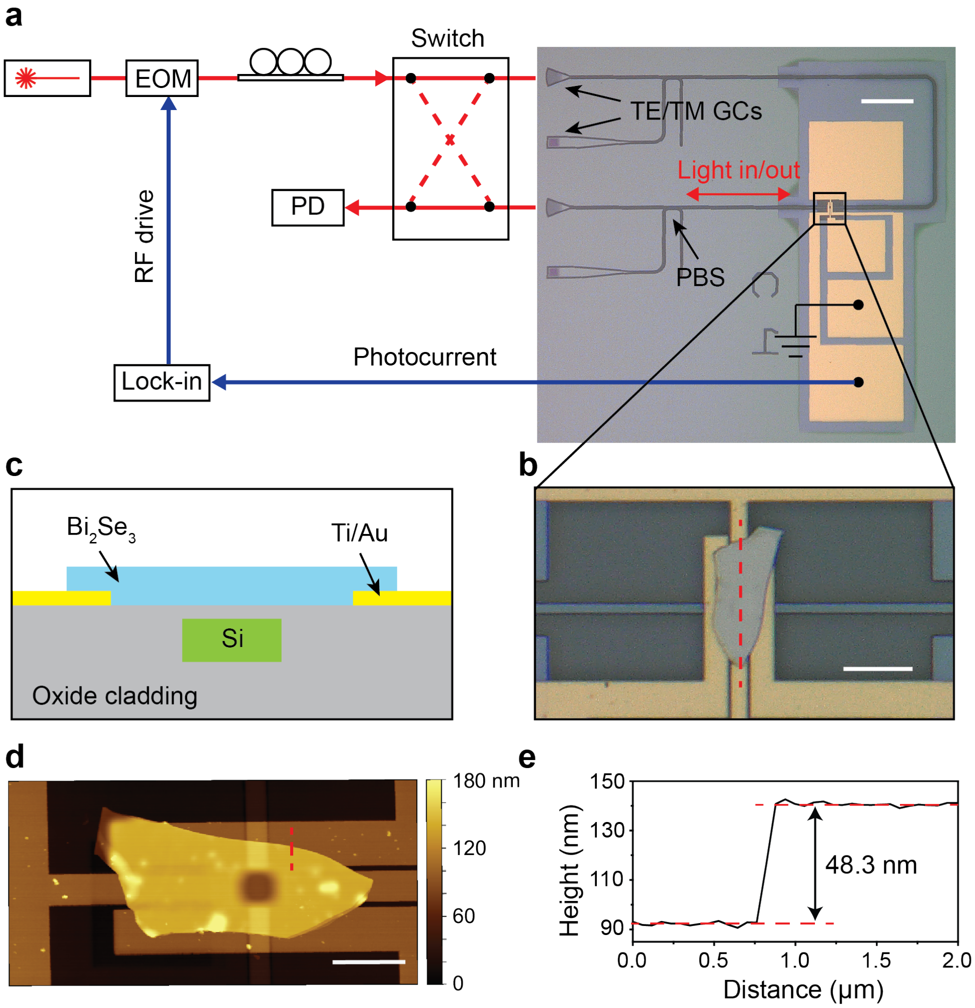

Supplementary Figure 1. Waveguide-integrated device layout and measurement scheme. **a**, The measurement setup. The CW laser source is modulated with an electro-optic modulator (EOM) and the induced photocurrent is detected by the lock-in amplifier. We couple TE/TM light into and out of the silicon waveguide through two pairs of grating coupler (GCs) and polarization beam splitters/couplers (PBS). The scale bar is 100 μm. **b**, Zoom-in view of the Bi_2Se_3 flake in Device C. The scale bar is 10 μm. **c**, The cross-section schematics shows the Bi_2Se_3 flake sitting on top of the cladding oxide to interact with the waveguide evanescent fields and the induced transverse current at the bottom surface being collected by a pair of contacts. **d**, AFM image of the same sample. The scale bar is 5 μm. **e**, The flake thickness is determined to be 48.3 nm with AFM.

Supplementary Note 1. Dependence of photocurrent response on optical power

We make sure the measured photoresponse on all devices are in the linear regime by measuring the photocurrent as a function of optical power. For example, Supplementary Figure 2 shows the result measured using the free-space optics setup on the device presented in the main text under various optical power excitations (α=0°, φ=0°), which shows a clear linear dependence on laser intensity. Therefore, the leading term of the photocurrent is of the second order in the oscillating electric field amplitudes and can in general be written as[1-3]

$$j_\lambda = \sigma_{\lambda\mu\nu}(\omega, \mathbf{k}) E^*_\mu(\omega) E_\nu(\omega) \quad (1)$$

where the optical conductivity $\sigma_{\lambda\mu\nu}$ is a third rank tensor, ω and $\mathbf{k}$ denote optical frequency and optical linear momentum, respectively. By performing a full symmetry analysis and considering the crystalline structure of Bi_2Se_3, the photocurrent perpendicular to the plane of light incidence can be derived to be of the form[1-3]

$$j_\mathrm{p}(\varphi, \alpha) = C(\varphi) \sin 2\alpha + L_1(\varphi) \sin 4\alpha + L_2(\varphi) \cos(4\alpha) + D(\varphi) \quad (2)$$

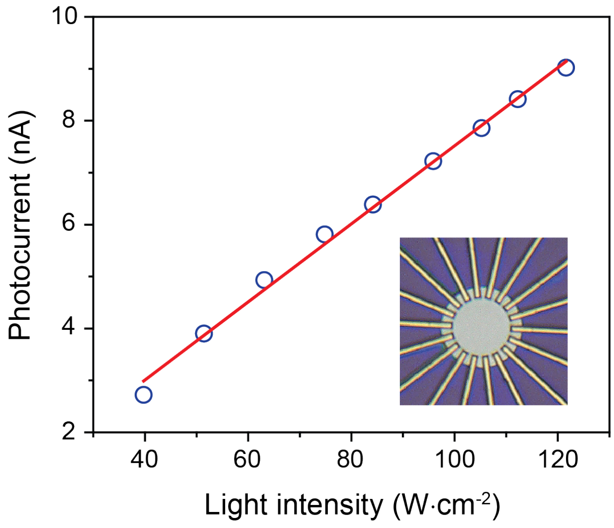

Supplementary Figure 2. Linear dependence of photocurrent on laser intensity. The polarization dependent photocurrent is measured using the free-space optics setup on the device presented in the main text (Fig. 2). The photocurrent shows a clear linear dependence on the laser intensity. The red line is the linear fitting to the experimental data.

Supplementary Note 2. Frequency responses of different photocurrent contributions

Different mechanisms (thermoelectric effect and photogalvanic effect) that contribute to the photocurrent have distinct frequency responses, which are measured in the free-space optics configuration shown in Fig. 2 in the main text. Supplementary Figure 3a shows the optical image of a device with a pair of metal contacts in the transverse direction (along y-axis). To isolate the CPGE contribution from the total photocurrent, we position the sample at the beam center (y=0) and measure the frequency-dependent photoresponse under the excitation of either left or right-handed circularly polarized light (α=45°,135°). According to Supplementary Equation 2, the thermoelectric (due to misalignment) and LPGE current are helicity-independent, whereas CPGE current changes sign for opposite light helicity. Therefore, by subtracting the photocurrent of the two measurements, only the CPGE contribution remains, showing a characteristic frequency of f_{3dB}=4 MHz (Fig. S3b). On the other hand, the thermoelectric photoresponse can also be isolated by measuring the photocurrent at positions shifted from the beam center (y=±25 μm) under linearly-polarized light excitation (α=0°). The thermoelectric contribution in the two measurements changes polarities due to the reversed temperature gradients, whereas the photogalvanic current remains unchanged. The difference of the two measurements gives the thermoelectric current and exhibits a characteristic frequency of f_{3dB}=30 kHz, much slower than that of the photogalvanic current. Therefore, we conclude that the photocurrent observed in the waveguide-integrated devices is dominated by the photogalvanic effects as the measured characteristic frequencies (Fig. 4a in the main text) are above 1 MHz.

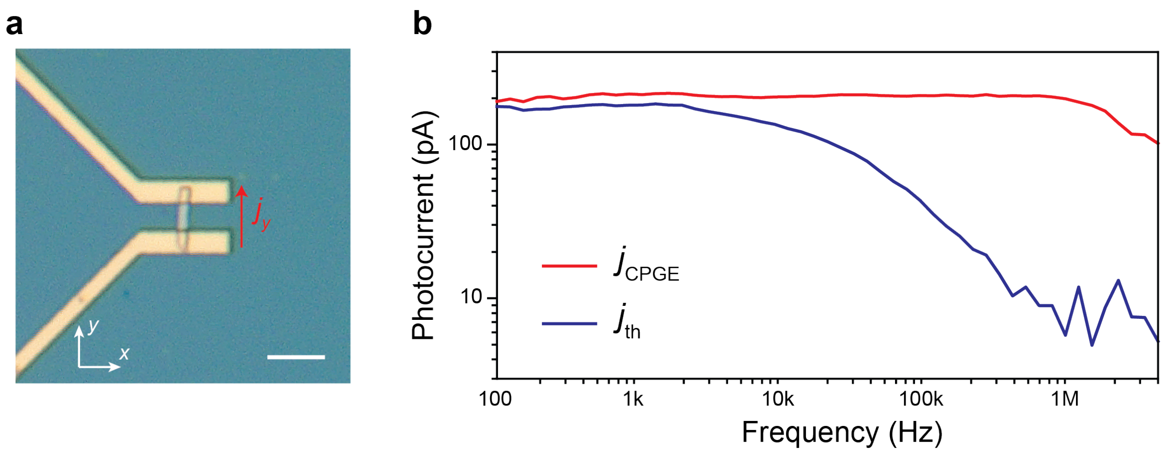

Supplementary Figure 3. Frequency responses of the thermoelectric and photogalvanic current. **a**, Optical microscopic image of a device used to determine the frequency response. The scale bar is 5 μm. **b**, The extracted CPGE and thermoelectric current show a cut-off frequencies of 4 MHz and 30 kHz, respectively.

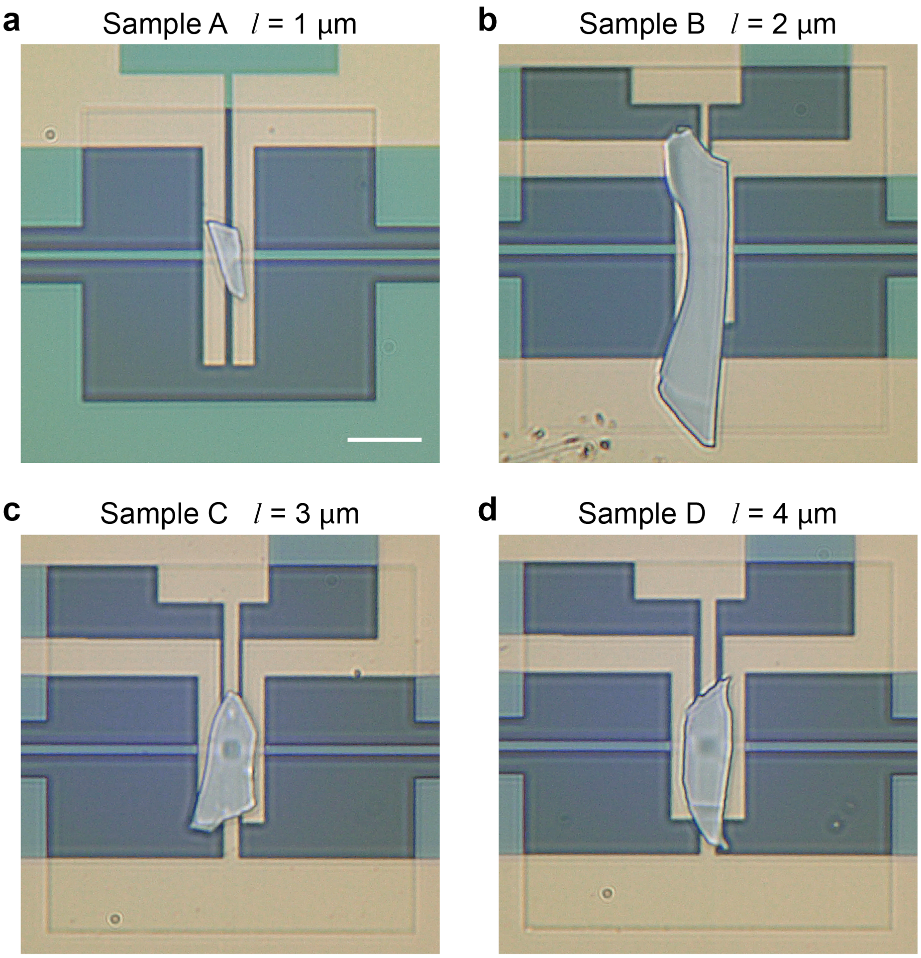

Supplementary Figure 4. Optical microscopic images of the TI-waveguide integrated devices. **a-d,** To study the spin-momentum locked interaction between waveguide mode and topological surface electrons, we have fabricated a series of devices with channel length varies from 1 to 4 μm. The corresponding photoresponse results are summarized in Fig. 4b in the main text. The scale bar is 10 μm.

Supplementary References

1 McIver, J. W., Hsieh, D., Steinberg, H., Jarillo-Herrero, P. & Gedik, N. Control over topological insulator photocurrents with light polarization. *Nat Nanotechnol* **7**, 96-100, doi:10.1038/nnano.2011.214 (2012).

2 Ganichev, S. D. & Prettl, W. Spin photocurrents in quantum wells. J. Phys. Condens. Matter 15, R935–R983 (2003).

3 Junck, A. Theory of Photocurrents in Topological Insulators. Ph.D. thesis, im Fachbereich Physik der Freien Universitat Berlin eingereichte (2015).
